# Enhancing Ground Beef Shelf Life Using *Psidium guajava* Leaf and *Oliveria decumbens* Essential Oils: Antimicrobial, Antioxidant, and Quality Preservation Effects Under Refrigeration

**DOI:** 10.1002/fsn3.72191

**Published:** 2026-08-03

**Authors:** Porandokht Barkhordari, Siavash Maktabi, Maryam Ghaderi Ghahfarokhi, Ali Heshmati

**Affiliations:** ^1^ Department of Food Hygiene, Faculty of Veterinary Medicine Shahid Chamran University of Ahvaz Ahvaz Iran; ^2^ Department of Food Science and Technology, National Nutrition and Food Technology Research Institute, Faculty of Nutrition Sciences and Food Technology Shahid Beheshti University of Medical Sciences Tehran Iran

**Keywords:** antimicrobials, antioxidants, food preservation, ground beef, *Oliveria decumbens*, *Psidium guajava*

## Abstract

This study evaluated *guava leaf* essential oil (GL‐EO) and flowers and seeds of *Oliveria decumbens* essential oil (*Od*‐EO) for their antimicrobial, antioxidant and sensory effects in ground beef. The essential oils were applied individually and in combination to ground beef samples (control, GL‐EO, *Od*‐EO, and GL‐EO + *Od*‐EO), which were packaged in sterile polyethylene zip‐lock bags and stored at 4°C for 10 days. In vitro analyses revealed concentration‐dependent antimicrobial activity against both Gram‐positive and Gram‐negative pathogens, with *Od*‐EO showing superior efficacy attributed to its high content of thymol and carvacrol. Antioxidant assays demonstrated strong radical scavenging capacity for both oils, with *Od*‐EO exhibiting 1.5‐fold higher activity in 2,2‐diphenyl‐1‐picrylhydrazyl (DPPH) assays compared to GL‐EO (*p* < 0.05). In ground beef applications, microbiological analyses (total mesophilic/psychrotrophic counts, coliforms and fungi) showed that both oils significantly (*p* < 0.05) inhibited microbial growth, with the GL‐EO + *Od*‐EO combination exhibiting notable preservative effects (approximately 2 log CFU/g reduction compared to controls). Chemical analyses revealed 45% lower thiobarbituric acid reactive substances (TBARS) values in treated samples, total volatile basic nitrogen (TVB‐N) levels maintained at < 20 mg/100 g throughout and improved storage, pH stability (6.3–6.5 vs. 6.8 in controls) (*p* < 0.05). Sensory evaluation indicated that the combined treatment received the highest acceptability scores for color, odor, texture, and overall acceptability, without negative sensory impact. The GL‐EO treatment also achieved high scores in preserving odor and color. These effects are associated with the oils' bioactive compounds (thymol, carvacrol, limonene, β‐caryophyllene). These results suggest that these essential oils, particularly in combination, can serve as effective natural preservatives, extending shelf‐life while maintaining quality. All treatments significantly extended shelf‐life by 4 days compared to control (*p* < 0.05).

## Graphical abstract

*Guava leaf* essential oil (GL‐EO) and *Oliveria decumbens* essential oil (*Od‐*EO) improved the microbial and oxidative stability of refrigerated ground beef and helped extend its shelf life. GL‐EO better preserved odor and color, while the combined GL‐EO + *Od*‐EO treatment provided the best texture preservation and highest overall acceptability.

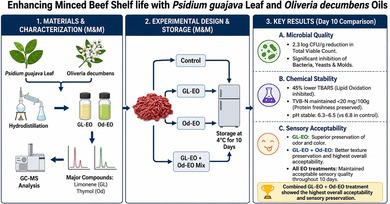

## 1 Introduction

The increasing consumer demand for clean‐label and naturally preserved foods has driven the search for effective plant‐based alternatives to synthetic preservatives (Biswas et al. 2023). This is particularly critical for highly perishable products like ground beef, which are susceptible to rapid quality deterioration due to lipid oxidation and microbial contamination by pathogens such as *Staphylococcus aureus* , *Escherichia coli* , and *Salmonella* spp. during refrigerated storage (Holman et al. 2020).

Several studies have reported the application of plant‐derived essential oils as natural preservatives in meat products due to their antimicrobial and antioxidant properties. Various essential oils have been shown to inhibit microbial growth and lipid oxidation in ground meat during refrigerated storage (Burt 2004). Recent research has also indicated that combining essential oils can enhance preservative efficacy through interactive or complementary effects.

*Psidium guajava* (guava) leaf is a promising candidate, recognized for its rich phytochemical profile. Its significant antimicrobial activity against common meat spoilage pathogens and its antioxidant capacity, attributed to compounds like flavonoids and tannins, have been well‐documented (Efoli‐Bam et al. 2024; Kasem et al. 2024). Studies confirm its effectiveness in reducing lipid oxidation in meat models (Sadiq et al. 2023; Nascimento et al. 2024).

Similarly, *Oliveria decumbens*, an ancient medicinal plant from the Umbelliferae family, is native to the warm regions of southern and western Iran and is widely used in traditional medicine. Its flower and seed essential oil has documented preservative potential (Amin et al. 2005; Nikravan et al. 2020).

Although previous studies have established the preservative potential of various essential oils in meat systems, significant research gaps remain. First, despite its known properties, the specific essential oil composition of Iranian *P. guajava* leaves and its direct application in food preservation are unreported. More importantly, limited information is available regarding the application of guava leaf essential oil (GL‐EO) and *Oliveria decumbens* flower and seed essential oil (*Od*‐EO), particularly in combination, in ground beef preservation.

Therefore, this study aimed to evaluate and compare the individual and combined effects of GL‐EO and *Od*‐EO on extending the shelf life of refrigerated ground beef. The specific objectives were to assess their impact on antimicrobial activity, antioxidant capacity, key preservation indices, and sensory attributes during storage. This research addresses a clear gap in knowledge and contributes to developing effective natural preservation strategies for the meat industry based on the combined use of essential oils.

## 2 Material and Methods

### 2.1 Collection and Extraction of Guava Leaf Essential Oil and *Oliveria decumbens* Essential Oil

Leaves of yellow guava ( *Psidium guajava* var. pomifera) were harvested from orchards in Rask County, Sistan and Baluchestan Province, Iran, a primary guava production region. Specimens of flowers and seeds of *Oliveria decumbens* were collected at full flowering stage from northeastern Khuzestan Province. Collection protocols were approved by the Sistan and Baluchestan Agricultural Organization and Shahid Chamran University of Ahvaz, Faculty of Agriculture. Plant materials were shade‐dried at ambient temperature (25°C ± 2°C) for 7 days. Essential oils (EOs) were extracted via hydrodistillation for 3 h using a Clevenger‐type apparatus. The essential oil yield (%) was calculated based on the dry weight of the plant material using the following formula:

$$\begin{array}{c} \mathrm{EO}\;\text{yield}\;\left(\%\right)\\ =\left(\text{Weight of extracted essential oil}/\text{Weight of}\;\mathrm{dry}\;\text{plant material}\right)\times 100 \end{array}$$

The guava leaf essential oil (GL‐EO) and *Oliveria decumbens* essential oil (*Od*‐EO) were dehydrated with anhydrous sodium sulfate (Na_2_SO₄; Merck, Darmstadt, Germany) and stored at 4°C in amber vials until analysis. Chemical profiling was performed using an Agilent 5977B GC–MS system (Agilent Technologies, USA) equipped with an HP‐5MS capillary column (30 m × 0.25 mm ID, 0.25 μm film thickness; 5% phenyl methyl polysiloxane stationary phase) using helium carrier gas (1.1 mL/min, split ratio 1:100), injector temperature 250°C, and oven program: 60°C (1 min) → 250°C at 5°C/min → 250°C (2 min), with electron ionization at 70 eV. The chemical constituents of the essential oils were identified based on their mass spectra, retention times, and calculated retention indices by comparison with published literature data (Adams 2007; Cheng et al. 2014). All analyses were performed in triplicate.

The chemical composition of Oliveria decumbens essential oil used in this study was previously characterized by Nikravan et al. (2020), and therefore was not repeated here to avoid redundancy.

#### 2.1.1 Previous Characterization of *Oliveria decumbens* Essential Oil (*Od*‐EO)

The antibacterial and antioxidant activities of *Oliveria decumbens* essential oil (*Od*‐EO) used in the present study were previously evaluated by Nikravan et al. (2020) as a joint work with our research at the same laboratory (Shahid Chamran University of Ahvaz, Faculty of Veterinary Medicine, Food Hygiene Laboratory). The same essential oil batch was used in both studies, which supports the consistency and comparability of the results. Standard in vitro assays, including disk diffusion agar, minimum inhibitory concentration (MIC), minimum bactericidal concentration (MBC), DPPH (2,2‐diphenyl‐1‐picrylhydrazyl), and ABTS (2,2′‐azino‐bis, 3‐ethylbenzothiazoline‐6‐sulfonic acid) assay were the same as this joint work. Therefore, these analyses were not repeated in the present study, and the published data (Nikravan et al. 2020) are used herein for comparative and contextual discussion purposes.

### 2.2 Determination of Antibacterial Activity of Essential Oils

The antibacterial activity of GL‐EO was evaluated against *Escherichia coli* O157:H7 (ATCC 25922), *Staphylococcus aureus* (ATCC 6538), *Salmonella Enteritidis* (ATCC 13076), and *Listeria monocytogenes* (ATCC 19118). Antibacterial assessment was carried out using the agar disc diffusion method, along with the determination of MIC and MBC, following standard microbiological procedures. The antibacterial activity of *Od*‐EO was previously characterized (Nikravan et al. 2020; see Section 2.1.1).

#### 2.2.1 Agar Disc Diffusion Method

An aliquot of bacterial suspension (10^8^ CFU/mL) was spread on the surface of brain heart infusion (BHI) agar (Condalab, Spain) using the surface‐spreading technique. A sterile paper disc impregnated with 20 μL of GL‐EO was placed on the BHI agar plates. The plates were incubated at 37°C for 24 h. Positive controls consisted of antibiotics (Ampicillin and Oxytetracycline, each 10 μg/disk) (Padtan Teb, Iran), and negative controls included BHI media containing dimethyl sulfoxide (DMSO, 0.5% vol/vol, Merck, Germany). The diameter of the inhibition zones formed around the discs was measured to determine the antibacterial activity of the essential oil. Brain heart infusion (BHI) agar was used instead of Mueller–Hinton agar because this assay aimed to evaluate the antibacterial activity of plant essential oils rather than perform standard antibiotic susceptibility testing. BHI agar provides better bacterial growth and facilitates the diffusion of hydrophobic essential oil compounds, as reported in previous studies (Hulankova et al. 2022; Raeisi et al. 2016).

#### 2.2.2 Determination of MIC and MBC

We selected Brain Heart Infusion (BHI) broth and agar for the MIC and MBC determination because its rich composition is known to better support the growth of fastidious bacteria and, crucially, facilitate the effective diffusion of the lipophilic GL‐EO, leading to more accurate results compared to the standard CA‐MHB and MHA (Hulankova et al. 2022). MIC and MBC were determined using double broth dilution of GL‐EO (0.015–4 μL/mL). At first, GL‐EO was dissolved in BHI (Merck, Germany) broth supplemented with 0.1% DMSO. Then, 180 μL of the broth containing various concentrations of GL‐EO was distributed into each well of a 96‐well polystyrene microtiter plate, and 20 μL of the bacterial culture (~10^6^ CFU/mL) was added to each well. After incubating the plates (37°C for 24 h), the lowest concentration of GL‐EO that inhibited the visible growth of the bacteria was determined as the MIC. The wells with the lowest concentration of GL‐EO that showed no growth on BHI agar were recognized as the MBC. Sterile BHI without bacterial culture was used as the negative control (Brannan 2009).

The ratio of MBC to MIC (MBC/MIC ratio) was calculated for all tested compounds.

### 2.3 Determination of Antioxidant Activity of Essential Oils

The antioxidant activity of Od‐EO was previously characterized (Nikravan et al. 2020; see Section 2.1.1).

#### 2.3.1 Determination of DPPH Radical Scavenging Activity

The scavenging activity against the DPPH radical was determined according to the method described by Lee and Yen (2006) with slight modifications. GL‐EO samples at different concentrations (312.5, 625, 1250, 2500, and 5000 μg/mL) were prepared in ethanol. For each reaction mixture, 0.3 mL of the GL‐EO ethanolic solution was mixed with 2.7 mL of DPPH solution (0.1 mmol L^−1^, Merck, Germany). The reaction mixture was incubated in the dark for 30 min at 25°C. The end point of the reaction was indicated by a visible color change of the DPPH solution from deep violet to pale yellow, after which the absorbance was measured at 517 nm using ethanol as the blank.

The control consisted of 2.7 mL of DPPH solution mixed with 0.3 mL of ethanol, while butylated hydroxytoluene (BHT; Merck, Germany) was used as a positive control. The DPPH radical scavenging activity was calculated using the following equation:

$$\text{DPPH scavenging activity}\;\left(\%\right)=\left(\mathrm{AC}-\mathrm{AS}\right)/\mathrm{AC}\times 100$$

where AC is the absorbance of the control and AS is the absorbance of the sample. All analyses were performed in triplicate. The antioxidant activity was expressed as IC_50_, defined as the concentration of essential oil required to scavenge 50% of DPPH radicals.

#### 2.3.2 ABTS Radical‐Scavenging Assay

The ABTS radical‐scavenging assay was used to evaluate the antioxidant activity of GL‐EO. The ABTS• + cation was generated by reacting 7 mM ABTS solution with 2.45 mM potassium persulfate (Merck, Germany), and the mixture was stored in the dark at room temperature for 12–16 h prior to use. The resulting ABTS• + solution was diluted with methanol to obtain an absorbance of 0.70 ± 0.02 at 734 nm.

For each reaction mixture, 0.3 mL of GL‐EO ethanolic solution at different concentrations (312.5, 625, 1250, 2500, and 5000 μg/mL) was mixed with 2.7 mL of the diluted ABTS• + solution. The reaction mixture was incubated for 6 min at room temperature. The end point of the reaction was indicated by a visible decrease in the blue‐green color intensity of the ABTS• + solution, and the absorbance was then measured at 734 nm.

BHT was used as a positive control, while the ABTS• + solution without essential oil served as the control (Wołosiak et al. 2021). The ABTS radical scavenging activity (%) was calculated using the following equation:

$$\text{ABTS scavenging activity}\;\left(\%\right)=\left(\mathrm{AC}-\mathrm{AS}\right)/\mathrm{AC}\times 100$$

where AC is the absorbance of the control and AS is the absorbance of the sample. All analyses were performed in triplicate.

### 2.4 Preparation and Addition of Essential Oils to Ground Beef

For each replicate, 1.5 kg of fresh, never‐frozen ground beef (70% lean meat, 30% fat; combining thigh and sirloin cuts), was prepared and divided into 250 g portions under sterile conditions in a laminar flow hood. Each 250 g portion was assigned to one of four treatment groups: control (no essential oil), GL‐EO, *Od*‐EO, and GL‐EO + *Od*‐EO. All samples were prepared freshly on day 0 and were not frozen or stored prior to analysis prepared.

For individual treatments, GL‐EO and *Od*‐EO were applied at 1% (w/w), while in the combined treatment (GL‐EO + *Od*‐EO), each oil was applied at 0.5% (w/w). Concentrations were calculated relative to meat weight to ensure reproducibility. The selected levels were determined based on preliminary antimicrobial screening and sensory acceptability evaluation. In the preliminary assessment, various concentrations (0.5%, 1%, 1.5%, and 2% w/w) of each essential oil were evaluated for antimicrobial effect and sensory acceptance by 9 trained panelists using a 5‐point hedonic scale. The 1% concentration was selected as the maximum concentration that maintained acceptable sensory characteristics while providing effective antimicrobial activity. All treatments were prepared in triplicate.

The essential oils were added dropwise using a sterile pipette and thoroughly mixed by hand for 2 min, followed by stomaching for 1 min to ensure uniform distribution throughout the meat matrix. Each sample was then packaged in sterile low‐density polyethylene (LDPE) zip‐lock bags (thickness: 0.05 mm) and stored at 4°C for subsequent chemical, microbiological, and sensory analyses on days 0, 2, 4, 6, 8, and 10 (Khaleque et al. 2016).

A positive control containing a standard chemical preservative was not included in this study, as the primary objective was to evaluate the efficacy of natural preservatives. Comparison with synthetic preservatives is recommended for future studies.

### 2.5 Fungal and Bacterial Counting Method

5 g of ground beef was placed in 90 mL of sterile normal saline in a stomacher bag and homogenized for 60 s using a stomacher. Serial dilutions and plating were performed according to standard protocols (APHA (American Public Health Association) 2012), and 100 μL of each dilution were spread on plate count agar (Merck, Germany). Petri dishes containing mesophilic bacteria were incubated at 37°C for 24 h, while those containing psychrotrophic bacteria were incubated at 7°C for 7–10 days. The pour plate method was used for coliform count. 1 mL of the desired dilutions was inoculated on violet red bile agar (Mirmedia, Iran) and plates were incubated at 37°C for 24 h. Presumptive red colonies were counted. For total fungal count, 0.1 mL of the desired dilutions was spread on rose bengal chloramphenicol agar media (Hi‐Media, India) and incubated at 25°C for 5 days. Colonies were counted on the fifth day. Results were reported as colony‐forming units per gram (cfu/g).

### 2.6 Analysis of Chemical Characteristics

#### 2.6.1 Measurement of pH

Ground beef samples (10 g) were homogenized in 90 mL of distilled water. The pH of the filtered homogenate was measured at room temperature using a pH meter (AZ‐86555, Taiwan) (Nikravan et al. 2025).

#### 2.6.2 Determination of Thiobarbituric Acid Reactive Substances (TBARS)

The TBARS value was measured as an indicator of lipid oxidation using the colorimetric assay described by Chen and Yen (2007). 5 g of each sample was homogenized with 25 mL of 20% trichloroacetic acid (Scharlau, Spain) and 20 mL of distilled water, then centrifuged at 8000 rpm for 10 min. 3 mL of the supernatant was mixed with 3 mL of thiobarbituric acid (Merck, Germany) reagent (0.02 M TBA in 90% acetic acid) in a glass tube. The tube was heated in a water bath at 90°C for 1 h and then cooled. The absorbance (As) of the sample and the absorbance (Ab) of the reagent were measured at 532 nm using a UV–Vis spectrophotometer (Cecil Instruments, UK), with distilled water as the blank. The TBARS value was expressed as mg of malondialdehyde (MDA) per kg of ground beef sample, using the following equation:

$$\text{TBARS}=\left[\left(\mathrm{As}-\mathrm{Ab}\right)\times 50\right]/200$$

#### 2.6.3 Measurement of Total Volatile Basic Nitrogen (TVBN)

The TVBN content was determined using the microdiffusion assay as described by Raeisi et al. (2016). In brief, 2 g of MgO and 2–3 drops of silicone were added to a mixture of 5 g of meat sample and 60 mL of distilled water, then transferred to tubes for distillation using a Kjeldahl apparatus. The distillate was absorbed in 40 mL of boric acid solution containing a mixed indicator (0.1 g methyl red and 0.1 g methyl blue dissolved in 100 mL of absolute ethanol). The boric acid solution was titrated with 0.1 N hydrochloric acid (HCl). The TVBN content was calculated using the following equation:

$$\text{TVBN}\;\left(\mathrm{mg}/100\;g\right)=\left(V\times C\times 14\times 100\right)/5$$

where V is the volume of HCl used, and C is its concentration.

### 2.7 Sensory Analysis

Sensory evaluation was conducted by a trained panel of nine assessors (five females and four males), aged 25–35 years (mean age: 29.5), who were students of Food Science and familiar with meat quality attributes. The same panelists participated in all sensory sessions throughout the storage period. Prior to participation, written informed consent was obtained from all panelists, and the sensory evaluation procedure was conducted in accordance with the institutional guidelines of the Department of Food Science.

Sensory evaluation was performed on days 0, 2, 4, 6, 8, and 10 of storage at room temperature under controlled conditions. Sensory sessions were conducted in triplicate at each sampling time. Samples (approximately 20 g each) were presented in odorless disposable containers and coded with random three‐digit numbers to avoid bias. Panelists evaluated odor, color, texture, and overall acceptability using a 5‐point hedonic scale. The scale ranged from 5 (no discoloration/extremely desirable/firm) to 1 (extreme discoloration/extremely unacceptable or off‐odors/very soft). Overall acceptability was calculated as the average of color, odor, and texture scores with equal weighting (Djenane et al. 2012). Samples with scores below 3 were considered unacceptable.

All sensory panelists voluntarily participated in the study, and informed consent was obtained prior to evaluation. The sensory protocol was conducted in accordance with institutional ethical guidelines and was determined to be exempt from formal ethical approval as it involved healthy adult volunteers evaluating food products with no health risk. All participants were fully aware of the nature of the study, and hygienic and laboratory safety standards were strictly followed throughout the sensory assessment process.

### 2.8 Statistical Analysis

All experiments were conducted in triplicate (*n* = 3). Sensory evaluation was performed by nine panelists (*N* = 9). Prior to applying parametric tests, the assumptions of normality and homogeneity of variance were assessed using the Shapiro–Wilk test and Levene's test, respectively. Both assumptions were met for all datasets (*p* > 0.05). Quantitative data were analyzed using one‐way analysis of variance (ANOVA), followed by Tukey's post hoc test to compare mean values among treatments. For storage studies, the effects of treatment and storage time were evaluated using two‐way repeated measures ANOVA (treatment × storage time), followed by Tukey's post hoc test. Sensory evaluation data, being non‐parametric, were analyzed using the Kruskal–Wallis test to compare treatments and the Friedman test to assess changes in sensory attributes during storage. All statistical analyses were performed using IBM SPSS Statistics version 22.0 (IBM Corp., Armonk, NY, USA); statistical significance was set at *p* < 0.05, and results are expressed as mean ± standard deviation (SD). Graphs were generated using Microsoft Excel. Furthermore, a formal checkerboard assay or FICI (Fractional Inhibitory Concentration Index) was not conducted to definitively establish synergy or antagonism between the essential oils.

### 2.9 Shelf‐Life Assessment

Shelf‐life endpoint was defined as the first failure in microbial, chemical, or sensory parameters.

## 3 Results

### 3.1 GL‐EO and *Od*‐EO Composition

All major chemical constituents of GL‐EO and *Od*‐EO are presented as percentages of the total oil to provide quantitative information for each parameter and facilitate comparison for the readers. The chemical composition of GL‐EO and *Od*‐EO was analyzed using gas chromatography–mass spectrometry (GC–MS), with identification based on comparison of mass spectra and retention indices with published data (Adams 2007; Cheng et al. 2014). The yield of GL‐EO was 1.02% (w/w), while the yield of *Od*‐EO is to be 5.7% (w/w).

**FIGURE 1:**
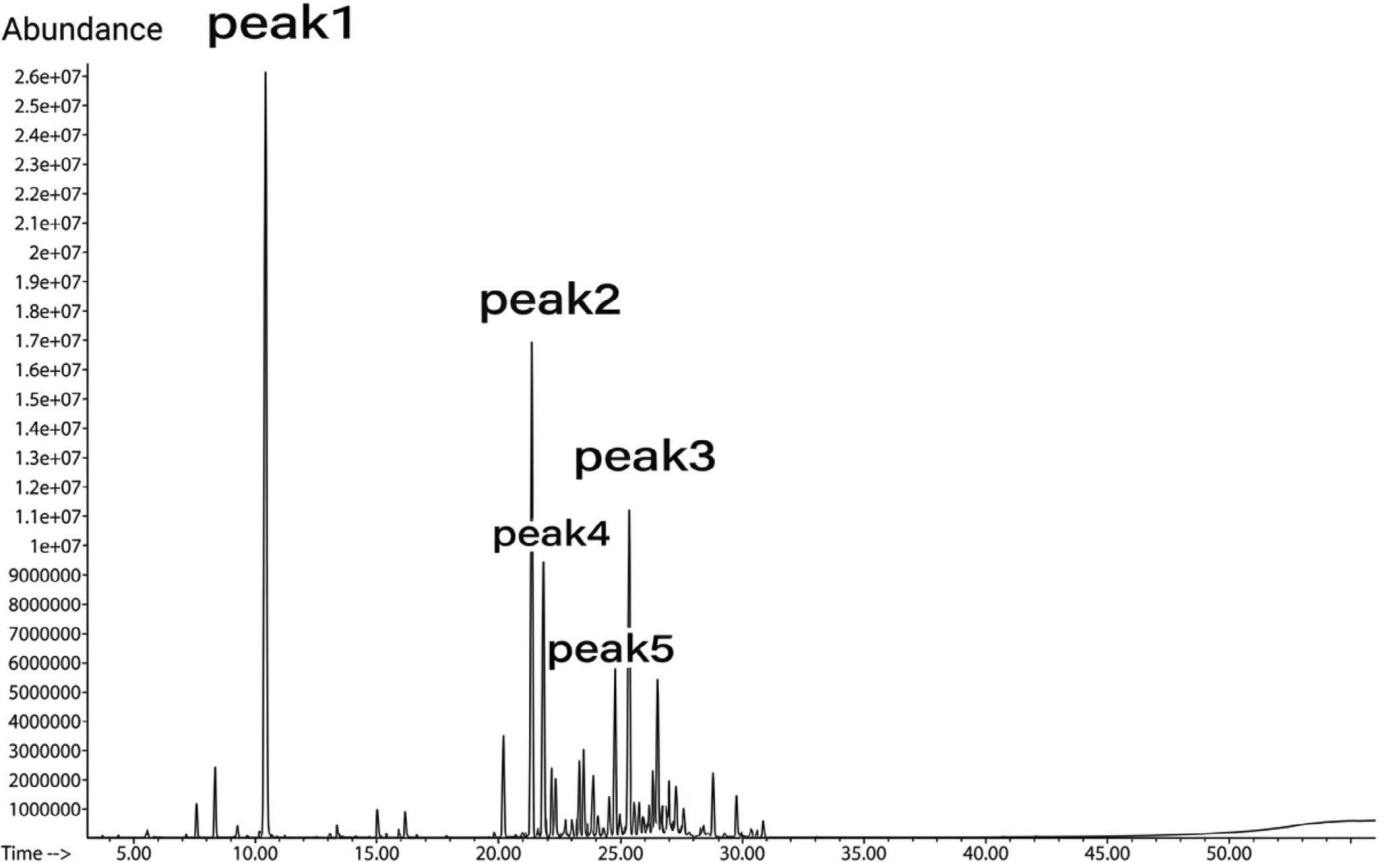
G*uava* leaf essential oil GC–MS: (Peak 1) Dl‐Limonene, (Peak 2) Trans‐Caryophyllene, (Peak 3) Nerolidol, (Peak 4) Globulol, (Peak 5) Caryophylladienol I (see Table 1).

A total of 42 chemical compounds were identified in GL‐EO. Limonene was the major component, comprising 20.36% of the total oil. Other significant components included trans‐caryophyllene (11.59%), globulol (10.91%), and nerolidol (6.73%) (Table 1; Figure 1). For *Od*‐EO, 15 chemical compounds were identified, accounting for 99.56% of the total oil. Thymol was the predominant compound (53.4%), followed by gamma‐terpinene (20.48%), p‐cymene (18.02%), and myristicin (2.7%). The full chemical composition of *Od*‐EO has already been reported. The major constituents of *Od*‐EO reported in the literature include thymol, γ‐terpinene, and p‐cymene (Nikravan et al. 2020).

**TABLE 1:** Chemical composition of Guava essential oil.

No	Compound	RT	RI	%
1	Alpha.‐Pinene	7.601	932	0.59
2	Benzaldehyde	8.345	938	1.33
3	Beta.‐Myrcene	9.266	986	0.22
4	Trans‐β‐Ocimene	10.222	1040	0.19
5	Dl‐Limonene	10.388	1018	20.36
6	Linalool	13.369	1139	0.36
7	P‐Mentha‐1(7),8‐Dien‐2‐ol	15.02	1184	0.57
8	Alpha. Terpineol	15.114	1181	0.15
9	Trans‐(+)‐ Carveol	15.910	1214	0.25
10	Carvacrol	16.16	1234	0.54
11	Neryl Acetate	19.82	1373	0.13
12	Alpha.‐Copaene	20.184	1396	2.13
13	Alpha.‐Gurjunene	21.059	1434	0.14
14	Trans‐Caryophyllene	21.340	1440	11.59
15	Zingiberene	21.534	1448	0.29
16	Beta.‐Gurjunene	21.632	1431	0.17
17	Aromadendrene	21.820	1456	6.08
18	Beta.‐Selinene	21.906	1501	0.43
19	Alpha.‐Humulene	22.175	1464	1.5
20	γ‐Muurolene	22.35	1509	1.34
21	Gamma.‐Gurjunene	22.627	1448	0.11
22	Alpha.‐Cadin a‐4,9‐Diene	22.725	1490	0.53
23	Beta.‐Selinene	22.982	1501	0.62
24	Alpha.‐Selinene	23.194	1505	0.47
25	Trans‐.AlphaBisabolene	23.331	1507	1.7
26	Beta. Bisabolene	23.486	1519	1.87
27	Gamma.‐Cadinene	23.646	1521	0.35
28	Cis‐ Calamenene	23.857	1523	1.37
29	Cadinadiene‐1,4	24.07	1538	0.58
30	Beta.‐Selinene	24.218	1501	0.11
31	Cis‐.Alpha.‐Bisabolene	24.287	1547	0.38
32	Nerolidol	24.790	1568	6.73
33	Spathulenol	25.179	1581	0.78
34	Globulol	25.339	1597	10.91
35	Veridiflorol	25.517	1599	2.69
36	Eremophilene	26.038	1610	1.65
37	Caryophylladienol I	26.52	1614	6.34
38	Tau.‐Muurolo	26.621	1624	0.98
39	Copaene	26.718	1651	0.83
40	Alpha.‐Cadinol	26.913	1662	2.64
41	Caryophyllenol II	27.27	1717	2.67
42	Isolongifolene	28.801	1750	1.78
	Total Constituents			94.45

*Note:* Mean values of three independent replicates.

Abbreviations: RI, Retention index; RT, Retention Time.

### 3.2 Microbiological Test Results of GL‐EO and *Od*‐EO

#### 3.2.1 Agar Disc Diffusion Method

**FIGURE 2:**
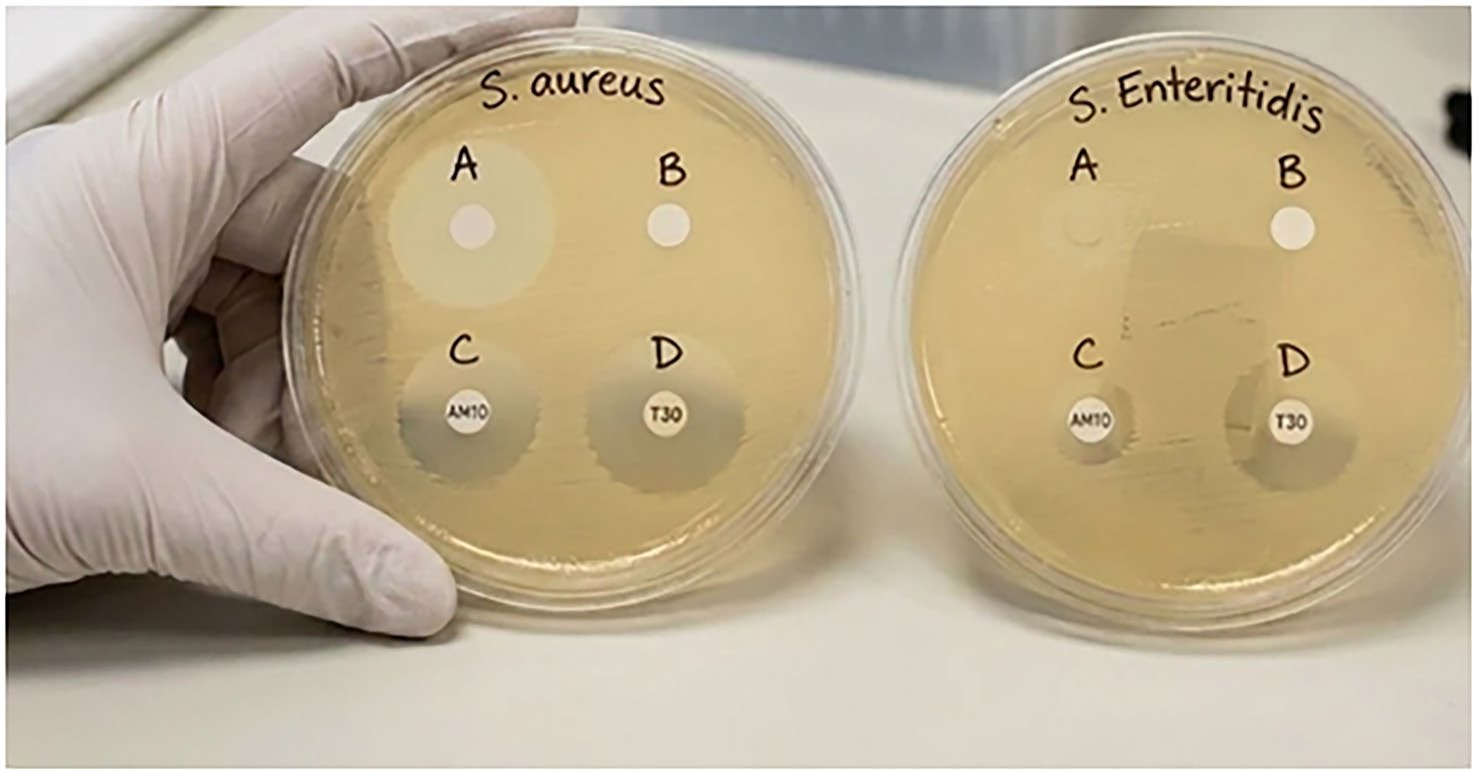
Disk diffusion assay for antimicrobial activity. Plates show inhibition zones around disks. (A) GL‐EO, (B) DMSO, (C) Ampicillin, (D) Oxytetracycline against *S. aureus an*d *S. enteritidis* .

The antibacterial activity of guava leaf essential oil (GL‐EO) against the tested microorganisms, as determined by the agar disc diffusion method, is presented in Table 2. Representative images of the inhibition zones formed by GL‐EO against the tested bacteria are shown in Figure 2. The results showed that Gram‐positive bacteria were significantly more sensitive to essential oils than Gram‐negative bacteria (*p* < 0.05). GL‐EO exhibited the strongest inhibitory effect against *Staphylococcus aureus* , forming an inhibition zone of 29.00 ± 1.0 mm, which was significantly larger (*p* < 0.05) than those formed against *Listeria monocytogenes* (20.66 ± 2.08 mm) and *Escherichia coli* O157:H7 (15.33 ± 1.52 mm). In contrast, *Salmonella Enteritidis* showed the lowest sensitivity, with an inhibition zone of only 9.66 ± 1.52 mm. Among the tested strains, *Staphylococcus aureus* exhibited the highest sensitivity, whereas *Salmonella Enteritidis* showed the lowest sensitivity to GL‐EO. These findings are consistent with the results obtained from the broth microdilution assay.

**TABLE 2:** Antimicrobial effects of *Od‐*EO and GL‐EO using disk diffusion method.

Microorganism	*Od*‐EO	Gentamicin	Ampicillin	DMSO
*S. aureus*	30.56 ± 1.91^a^	22.66 ± 1.24^c^	26.66 ± 0.47^b^	0.00 ± 0.00^d^
*L. monocytogenes*	19.73 ± 2.65^bc^	25.7 ± 2.47^ab^	29.56 ± 2.18^a^	0.00 ± 0.00^d^
*E. coli* O157:H*7*	13.86 ± 1.32^c^	19.76 ± 0.88^a^	14.53 ± 0.72^bc^	0.00 ± 0.00^d^
*P. aeruginosa*	11.43 ± 0.49^c^	17.76 ± 0.55^a^	0.00 ± 0.00^d^	0.00 ± 0.00^d^

	GL‐EO	Oxytetracycline	Ampicillin	DMSO
*S. aureus*	29.00 ± 1.0^c^	24.33 ± 0.57^c^	21.33 ± 1.52^b^	0.00 ± 0.00^d^
*L. monocytogenes*	20.66 ± 2.08^bc^	23.00 ± 1.00^bc^	22.66 ± 1.52^b^	0.00 ± 0.00^d^
*E. coli* O157:H7	15.33 ± 1.52^ab^	18.33 ± 1.52^ab^	17.33 ± 1.52^ab^	0.00 ± 0.00^d^
*S. enteritidis*	9.66 ± 1.52^a^	16.33 ± 1.15^a^	11.00 ± 1.00^a^	0.00 ± 0.00^d^

*Note:* Mean values of three replicates ± standard deviation (SD). Different superscript letters within the same row indicate significant differences (*p* < 0.05) based on one‐way ANOVA followed by Tukey's post hoc test. *Od*‐EO data were obtained from the previous study by Nikravan et al. (2020), whereas GL‐EO antimicrobial experiments were performed in the present study.

Abbreviations: DMSO, dimethyl sulfoxide; GL‐EO, guava leaf essential oil; *Od*‐EO, Oliveria decumbens essential oil.

#### 3.2.2 Determination of MIC and MBC

The minimum inhibitory concentration (MIC) and minimum bactericidal concentration (MBC) of GL‐EO and *Od*‐EO against the target foodborne pathogens were determined using the microdilution broth method, and the complete results are presented in Table 3. The MIC and MBC data for *Od*‐EO were derived from a previously published study (Nikravan et al. 2020) and are included here for comparative analysis.

**TABLE 3:** MIC and MBC (mg/mL) and MBC/MIC ratio of *Od‐*EO and GL‐EO against foodborne bacteria.

Microorganism	*Od*‐EO			GL‐EO		
	MIC	MBC	MBC/MIC ratio	MIC	MBC	MBC/MIC ratio
*S. aureus*	0.312	0.625	2	0.625	1.25	2
*L. monocytogenes*	2.5	5	2	2.5	2.5	1
*E. coli* O157:H7	10	10	1	80	> 80	> 1
*S*. Entrididis	—	—	—	20	40	2

*Note:* Mean values of three replicates ± standard deviation (SD). Different superscript letters within the same row indicate significant differences (*p* < 0.05). *Od*‐EO data were obtained from the previous study by Nikravan et al. (2020), whereas GL‐EO antimicrobial experiments were performed in the present study. MBC/MIC ratio, ratio of minimum bactericidal concentration to minimum inhibitory concentration (a value ≤ 4 indicates bactericidal activity).

Abbreviations: GL‐EO, guava leaf essential oil; MBC, minimum bactericidal concentration; MIC, minimum inhibitory concentration; *Od‐*EO, *Oliveria decumbens* essential oil.

As detailed in Table 3, Both essential oils demonstrated potent bactericidal activity against Gram‐positive pathogens. GL‐EO was most effective against *S. aureus* (MIC = 0.625 mg/mL; MBC = 1.25 mg/mL), followed by *L. monocytogenes* (MIC = MBC = 2.5 mg/mL). In contrast, Gram‐negative bacteria exhibited notably higher resistance. *E. coli* O157:H7 was highly resistant to GL‐EO (MIC = 80 mg/mL; MBC > 80 mg/mL), while *Salmonella* Enteritidis showed an MIC of 20 mg/mL and an MBC of 40 mg/mL for GL‐EO. *Od*‐EO showed a similar pattern, with the lowest MIC value against *S. aureus* (0.312 mg/mL).

The tolerance ratio (MBC ÷ MIC) was calculated for all bacterial strains to provide additional insight into their susceptibility. As shown in Table 3, the MBC/MIC ratios for all susceptible strains were either 1, 2, or > 1, all of which are below the established bactericidal threshold of 4. This confirms a marked bactericidal (rather than bacteriostatic) mode of action for both essential oils against the susceptible strains.

### 3.3 DPPH and ABTS Radical Scavenging Assays

The antioxidant activities of guava leaf essential oil (GL‐EO) and *Oliveria decumbens* essential oil (*Od*‐EO) were evaluated using both DPPH and ABTS radical scavenging assays, and the results are presented in Table 4.

**TABLE 4:** Antioxidant capacity of *Od‐*EO and GL‐EO using DPPH and ABTS^+^ methods.

Method	IC50 (μg/mL)		
	BHT	*Od‐*EO	GL‐EO
DPPH	18.57 ± 3.41^c^	1456.95 ± 109.99^a^	2399.11 ± 64.32^a^
ABTS	12.32 ± 0.37^c^	565.83 ± 25.05^a^	> 5000^b^

*Note:* Mean values of three replicates ± standard deviation (SD). Different superscript letters within the same row indicate significant differences (*p* < 0.05) based on one‐way ANOVA followed by Tukey's post hoc test. *Od*‐EO data were obtained from the previous study by Nikravan et al. (2020), whereas GL‐EO antimicrobial experiments were performed in the present study.

Abbreviations: ABTS, 2,2′‐azinobis‐ (3‐ethylbenzothiazoline‐6‐sulfonic acid) radical cation assay; DPPH, 2,2‐diphenyl‐1‐picrylhydrazyl radical scavenging assay; GL‐EO, Guava leaf essential oil; IC_50_, concentration required to inhibit 50% of radical activity; *Od*‐EO, Oliveria decumbens essential oil.

The quantitative results revealed a clear hierarchy of antioxidant potency. The synthetic antioxidant BHT showed the strongest activity, with IC₅₀ values of 18.57 ± 3.41 μg/mL in the DPPH assay and 12.32 ± 0.37 μg/mL in the ABTS assay. In comparison, both essential oils exhibited significantly (*p* < 0.05) lower radical scavenging activity. *Od*‐EO demonstrated moderate antioxidant capacity, with an IC₅₀ of 1456.95 ± 109.99 μg/mL in the DPPH assay and 565.83 ± 25.05 μg/mL in the ABTS assay. GL‐EO showed the weakest activity among the tested samples, with an IC₅₀ of 2399.11 ± 64.32 μg/mL in the DPPH assay. In the ABTS assay, its scavenging activity was particularly low, with an IC₅₀ value exceeding the highest concentration tested (5000 μg/mL), indicating a substantially lower antioxidant capacity compared to *Od*‐EO and BHT in this model system. Consequently, *Od*‐EO showed significantly higher (*p* < 0.05) antioxidant activity than GL‐EO in both assays (Table 4).

### 3.4 Microbial Analysis of Ground Beef Containing Essential Oils

**FIGURE 3:**
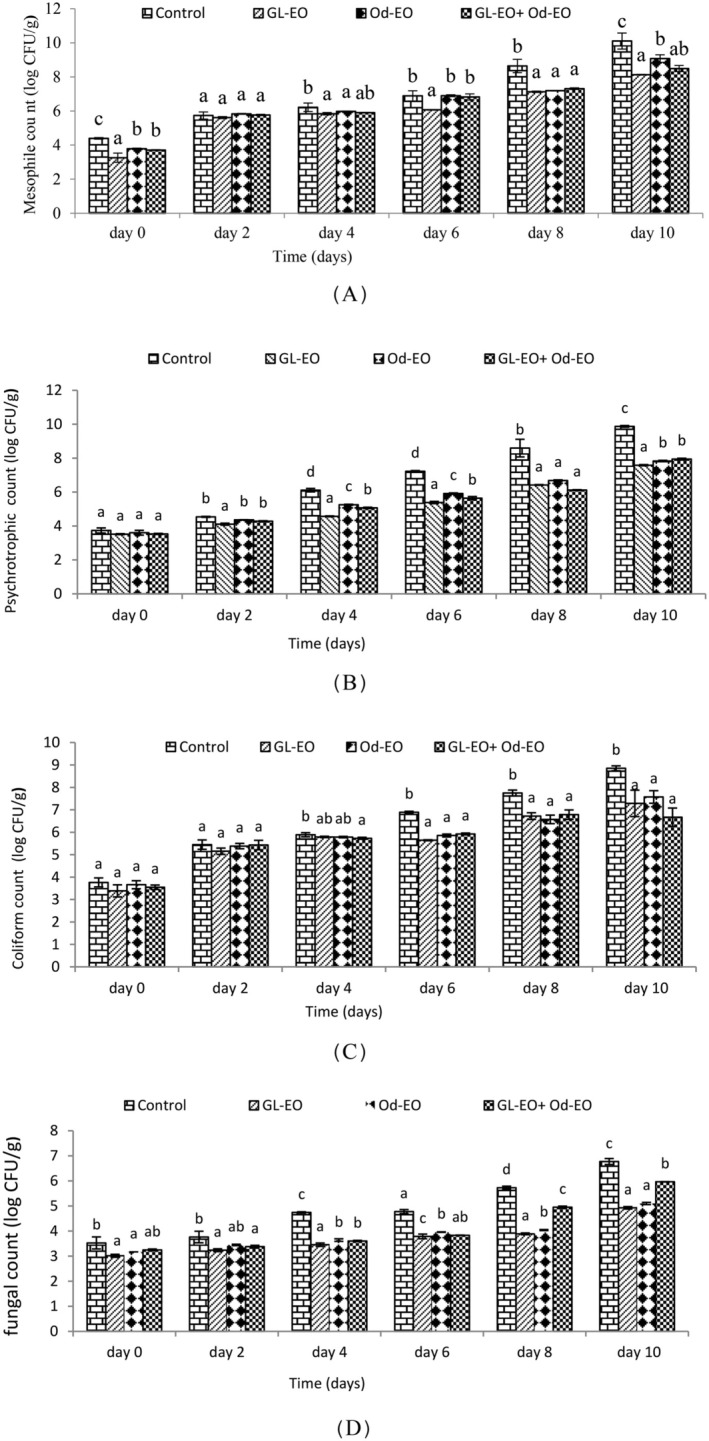
Changes in microbial counts of ground beef samples during 10 days of refrigerated storage: (A) total viable count, (B) psychrotrophic bacteria count, (C) coliform count, and (D) fungal count. Values are expressed as mean ± SD (*n* = 3). Different lowercase letters indicate significant differences (*p* < 0.05) between treatments at the same storage time, based on one‐way ANOVA followed by Tukey's post hoc test.

**FIGURE 4:**
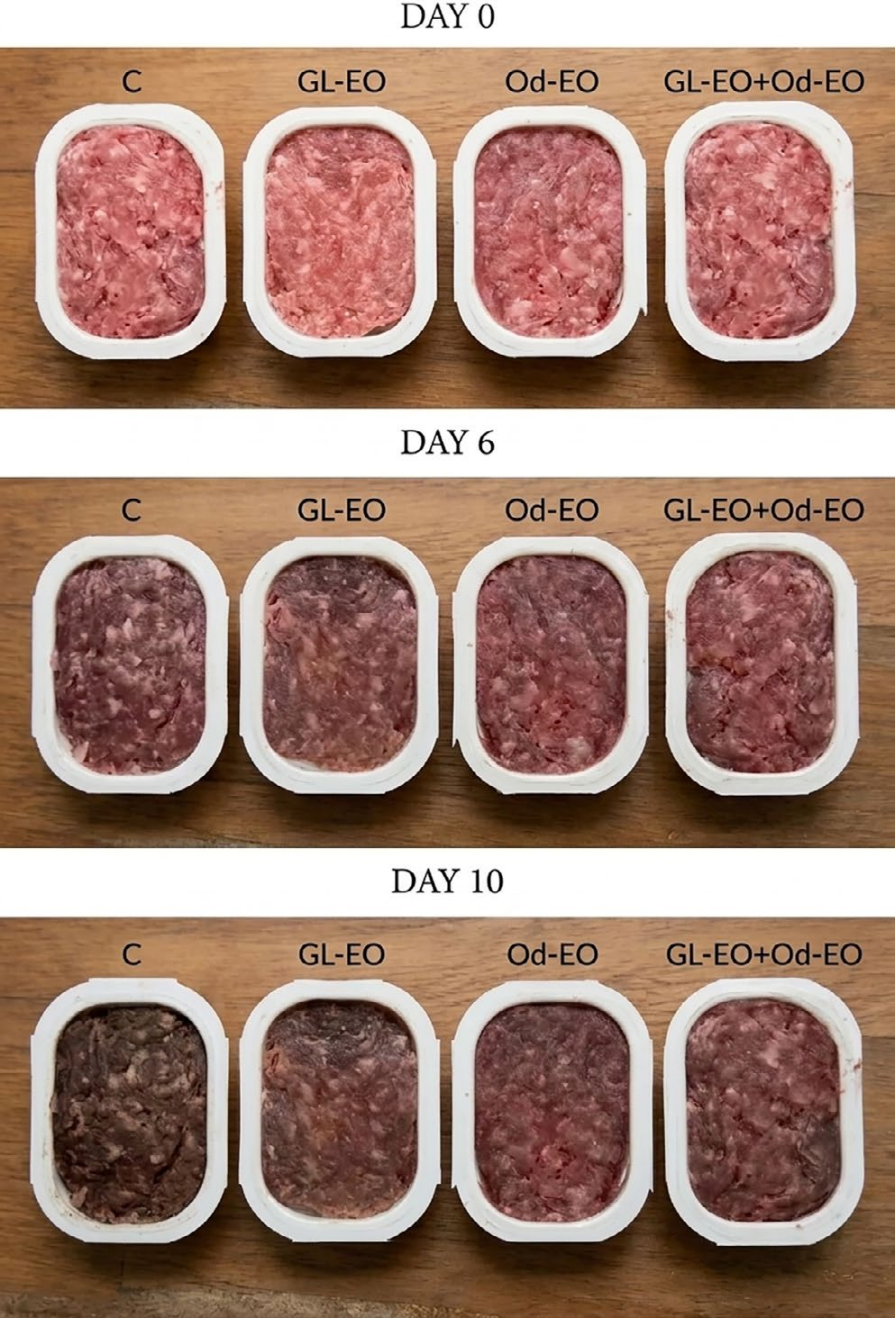
Visual appearance of ground beef samples at Days 0, 6, and 10 under different treatments: Control (C), *guava* leaf essential oil (GL‑EO), *Oliveria decumbens* essential oil (*Od*‑EO), and their combination (GL‑EO + *Od*‑EO).

Figure 3A–D illustrates the microbial analysis results of ground beef samples during 10 days of refrigerated storage. Visual changes in the appearance of beef samples at days 0, 6, and 10 are presented in Figure 4. A progressive increase in the total viable (mesophilic) bacterial count, psychrotrophic bacteria, coliforms, and fungi was observed in all treatments with increasing storage time. The control group exhibited the highest microbial counts on all sampling days. For instance, on day 10, the total viable count in the control reached 10.47 ± 0.47 log CFU/g, whereas treatments with GL‐EO, *Od*‐EO, and their combination significantly (*p* < 0.05) reduced these counts to 8.13 ± 0.02, 9.07 ± 0.22, and 8.49 ± 0.18 log CFU/g, respectively. A similar trend was observed for psychrotrophic and coliform counts (Figure 3B,C).

The combined treatment showed substantial preservative effects (~1.98 log CFU/g reduction). The GL‐EO treatment maintained a low microbial population (~2.34 log CFU/g reduction), while Od‐EO also contributed to microbial reduction (9.07 log CFU/g); all reductions were statistically significant (*p* < 0.05). This trend was consistent across all measured microbial groups, with the combination treatment also resulting in the lowest final counts for psychrotrophic bacteria (7.94 ± 0.05 log CFU/g) and coliforms (6.67 ± 0.41 log CFU/g) on day 10.

### 3.5 Chemical Analysis Results

**FIGURE 5:**
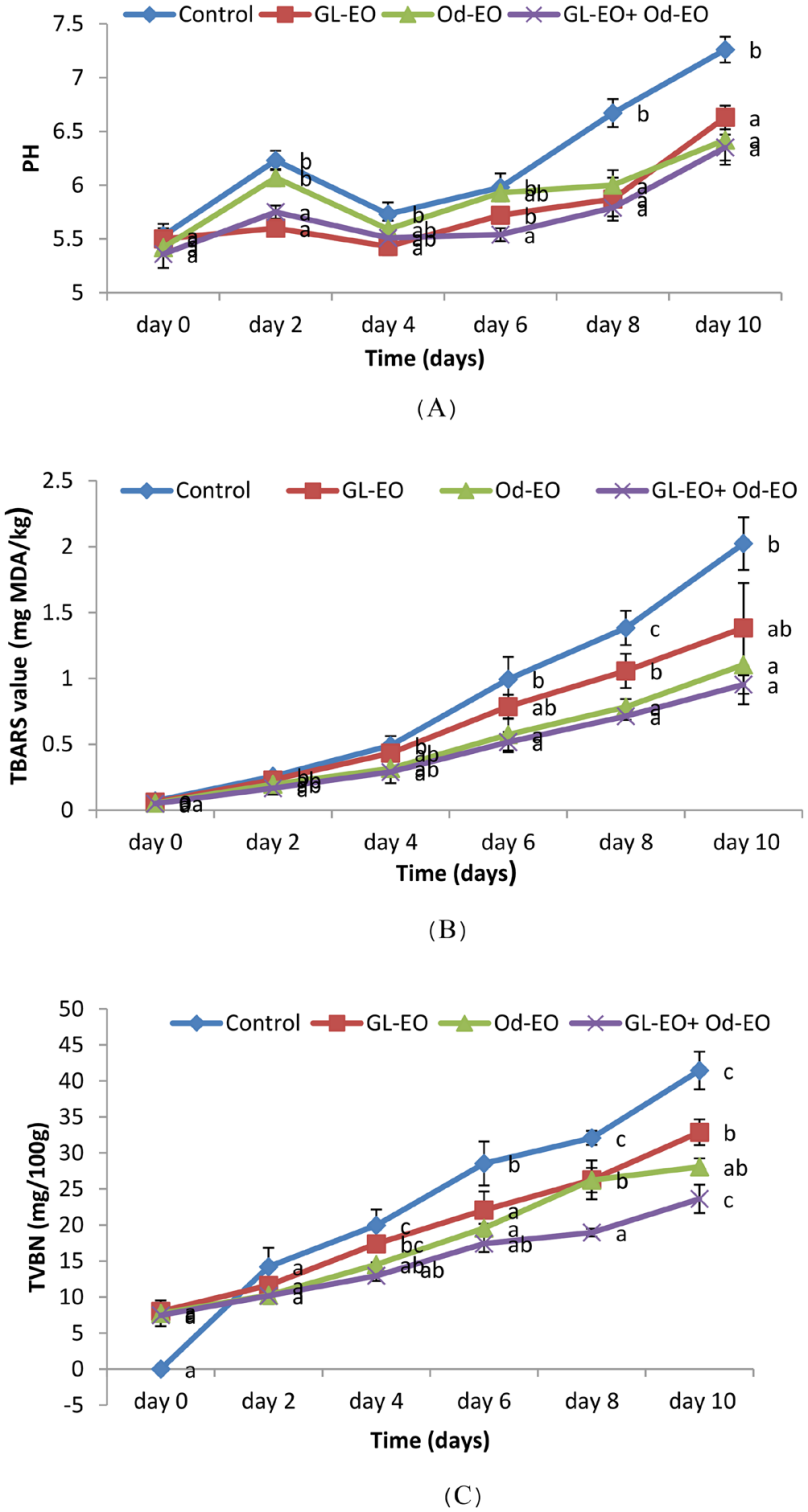
Chemical analysis (pH, TBA, and TVBN) of ground beef samples during 10 days of refrigerated storage. Values are expressed as mean ± SD (*n* = 3). Different lowercase letters indicate significant differences (*p* < 0.05) between treatments on the same day, based on one‐way ANOVA followed by Tukey's post hoc test.

Figure 5A–C presents the chemical analysis results of ground beef samples during 10 days of refrigerated storage. On day 0, significant differences (*p* < 0.05) in pH values were observed among the treatments, ranging from 5.36 ± 0.13 in the GL‐EO + *Od*‐EO combination to 5.53 ± 0.11 in the control. During storage, pH values increased in all treatments, with the lowest final pH recorded in the GL‐EO + *Od‐*EO combination on day 10 (6.35 ± 0.12), compared to the control (7.26 ± 0.12).

No significant differences (*p* > 0.05) in thiobarbituric acid (TBA) values were observed among treatments on day 0, with values between 0.04 ± 0.00 and 0.07 ± 0.01 mg MDA/kg. However, TBA values increased progressively during storage in all treatments, with the highest values observed in the control group, reaching 2.02 ± 0.2 mg MDA/kg on day 10, which was significantly higher (*p* < 0.05) than all essential oil treatments.

The total volatile basic nitrogen (TVBN) content increased gradually in all samples during refrigerated storage, indicating normal protein degradation over time. Nevertheless, the sample treated with the combined essential oils of GL‐EO and *Od*‐EO exhibited the lowest TVBN value (23.62 ± 1.97 mg/100 g) at the end of storage, which was significantly lower than the control group (41.44 ± 2.61 mg/100 g). This corresponds to an approximate 43% reduction in TVBN compared to the untreated samples, confirming the marked preservative and enhanced effect of the combined essential oils in suppressing the accumulation of volatile nitrogen compounds and delaying spoilage.

Although single‐oil treatments also resulted in moderate decreases compared with the control, their inhibitory impact was less pronounced than that of the combination treatment.

### 3.6 Sensory and Physical Changes

**FIGURE 6:**
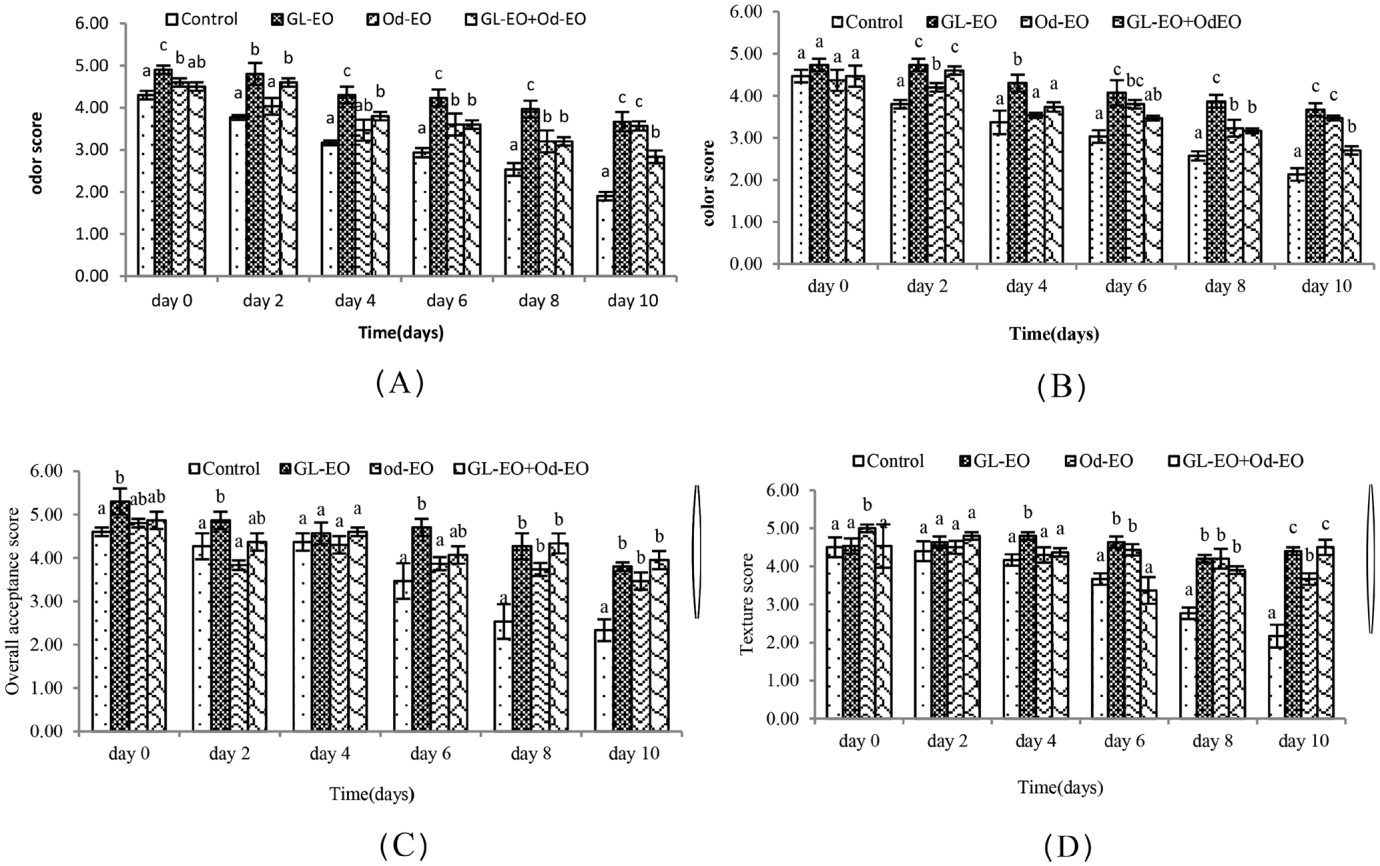
Sensory evaluation (A) odor, (B) color, (C) texture, and (D) overall acceptability of ground beef samples during 10 days of refrigerated storage. Different lowercase letters indicate significant differences (*p* < 0.05) between treatments at the same storage time, based on Kruskal–Wallis test.

Figure 6A–D depicts the sensory evaluation of minced meat samples stored at 4°C over 10 days, assessing odor, color, texture, and overall acceptability using a 5‐point hedonic scale (1 = extremely unacceptable, 5 = extremely desirable). A score below 3 was considered unacceptable.

Sensory analysis revealed that all attributes were significantly (*p* < 0.05) influenced by storage time and treatment. Odor quality declined rapidly in the control group, which fell below the acceptability threshold 2.93 ± 0.11 by day 6. In contrast, all essential oil treatments maintained acceptable odor scores throughout storage, with the guava leaf essential oil (GL‐EO) treatment showing the best performance 4.23 ± 0.20 on day 6.

Color was best preserved in the guava leaf essential oil (GL‐EO) treatment, which achieved the highest score of 3.87 ± 0.15. The combined GL‐EO + *Od*‐EO treatment also effectively delayed discoloration, maintaining a score of 3.17 ± 0.05 on day 8, which was significantly higher than the control 2.57 ± 0.11.

Texture degradation was most severe in the control, which became unacceptably soft 2.17 ± 0.30 by day 10. In contrast, the combined treatment retained a firmer texture, scoring 3.50 ± 0.20 on the final day.

Overall acceptability, calculated as the mean of odor, color, and texture scores, clearly reflected these trends. The control group became unacceptable by day 6 (2.93 ± 0.11) and reached a low of 2.33 ± 0.25 on day 10. The GL‐EO + *Od‐*EO combination demonstrated the highest preservation of sensory quality, maintaining an acceptable score of 3.95 ± 0.21 until the end of the storage period. These results confirm that the combined essential oil treatment effectively extends the sensory shelf‐life of refrigerated ground meat.

## 4 Discussion

### 4.1 Chemical Composition of Essential Oils

The chemical composition of essential oils is influenced by multiple factors, including plant growth stage, cultivar type, extraction methodology, and environmental conditions (e.g., climate, geography, and soil properties) (Saidi et al., 2014). In the present study, GL‐EO (guava leaf essential oil) was characterized by a high abundance of the monoterpene (DL‐limonene) and the sesquiterpenes (trans‐caryophyllene and globulol), which collectively accounted for 42.86% of the total oil composition.

These results are consistent with prior studies but also reveal notable variations. For example, Soliman et al. (2016) reported a predominance of sesquiterpenes in guava essential oil, whereas our findings indicate a higher relative concentration of monoterpenes, particularly DL‐limonene. This discrepancy could be attributed to differences in plant maturity at harvest or extraction techniques (hydrodistillation vs. solvent extraction) (Volpini‐Klein et al. 2021). Hassan et al. (2020) identified limonene as a major volatile constituent in Malaysian red and Indian white guava cultivars, while Egyptian varieties exhibited elevated β‐caryophyllene levels, a pattern that aligns with our detection of trans‐caryophyllene, its stereoisomer. The antimicrobial and antioxidant properties associated with β‐caryophyllene suggest that GL‐EO may share similar bioactivities, warranting further investigation (Hassan et al. 2020). Interestingly, Chaturvedi et al. (2021) reported higher concentrations of caryophyllene oxide (an oxidized derivative of β‐caryophyllene) in Indian guava leaves compared to our study. This divergence might reflect post‐harvest oxidative changes or genetic differences between guava populations (Bylappa and Nag 2024). Alam et al. (2021) similarly identified limonene and β‐caryophyllene as dominant constituents, reinforcing their ecological or physiological significance in guava. The strong antimicrobial effects of D‐limonene further highlight the potential of GL‐EO as a natural preservative or therapeutic agent (Alam et al., 2021). The specific chemical profile obtained in this study, therefore, appears to be a result of the combined influence of the plant's geographical origin, harvest maturity, and the employed hydrodistillation method. The observed compositional variations underscore the need for standardized protocols in essential oil research, the bioactivity of GL‐EO constituents (e.g., possible interactive effects between limonene and caryophyllene) should be experimentally validated, and comparative studies across cultivars and growing regions could elucidate genetic vs. environmental influences on oil profiles. Overall, the predominance of limonene and caryophyllene derivatives suggests that GL‐EO possesses promising antimicrobial and antioxidant potential, supporting its application as a natural preservative.

The chemical profile of *Oliveria decumbens* essential oil (*Od*‐EO) markedly differed from that of GL‐EO. In *Od*‐EO, the phenolic monoterpene, thymol (a potent antimicrobial agent), along with γ‐terpinene and p‐cymene (precursors in thymol biosynthesis), dominated the composition, collectively representing 91% of the total oil. These findings align closely with prior studies. For instance, Boveiri Dehsheikh et al. (2024) reported that oxygenated monoterpenes (particularly thymol, carvacrol, γ‐terpinene, p‐cymene, and myristicin) constituted the majority of *Od*‐EO, corroborating our results. Similarly, Karami et al. (2020) identified thymol, carvacrol, γ‐terpinene, and p‐cymene as major components, further supporting the consistency of these chemotypes across different populations.

The high concentration of thymol, a compound widely documented for its broad‐spectrum antimicrobial activity (Dong et al. 2024), suggests strong potential for *Od*‐EO in pharmaceutical or preservative applications. Notably, the presence of γ‐terpinene and p‐cymene (both biosynthetic precursors to thymol) may contribute to enhanced enhance bioactivity, as these compounds are known to improve membrane permeability in target microorganisms (Raikwar et al. 2024). While monoterpenes dominated *Od*‐EO, sesquiterpenes (though minor constituents) could further contribute to antimicrobial effects. As demonstrated by Karami et al. (2020), high sesquiterpene concentrations disrupt microbial ion transport and accumulate in bacterial membranes, leading to loss of structural integrity, cell lysis, and death (Karami et al. 2020). Overall, the thymol‐rich chemotype of *Od*‐EO explains its strong antimicrobial efficacy and supports its suitability for food preservation applications. The high consistency of this thymol‐dominant profile across studies, including ours, confirms the stability of this chemotype in Oliveria decumbens, making it a reliable source of potent natural antimicrobials.

This mechanism may explain the observed efficacy of *Od‐*EO against resistant pathogens in prior studies (Javid Moghadam et al. 2024).

### 4.2 Antimicrobial and Antioxidant Activity of GL‐EO and *Od*‐EO

The present study demonstrated that Gram‐positive bacteria were more susceptible to both GL‐EO and *Od‐*EO, as evidenced by agar disc diffusion and MIC/MBC assays. In particular, *S. aureus* and *L. monocytogenes* showed larger inhibition zones and lower MIC and MBC values compared to Gram‐negative bacteria. The MIC values for *S. aureus* were 0.312 mg/mL for GL‐EO and 0.625 mg/mL for *Od‐EO*, indicating strong antibacterial activity, whereas *E. coli O157:H7* exhibited markedly higher MIC values (10–80 mg/mL), reflecting greater resistance.

Similarly, the MBC/MIC tolerance ratios indicated a bactericidal effect of both essential oils against Gram‐positive bacteria, while a reduced efficacy was observed against Gram‐negative strains. The lower susceptibility of *E. coli* and *S. enteritidis* may be attributed to the presence of an outer membrane rich in lipopolysaccharides, which restricts the penetration of hydrophobic compounds such as essential oils. Comparable trends have been reported by Pateiro et al. (2021), who observed higher resistance of Gram‐negative bacteria to plant‐derived essential oils. The antimicrobial activity of GL‐EO observed in this study is consistent with previous reports showing its effectiveness against *S. aureus* , *Bacillus subtilis* , and *Streptococcus faecalis* (Soliman et al. 2016). Likewise, Eftekhari et al. (2019) reported that *Od‐EO* exerted strong inhibitory effects against *S. aureus* and *S. epidermidis* , while *Pseudomonas aeruginosa* exhibited minimal sensitivity, in agreement with the resistance pattern observed for Gram‐negative bacteria in the present study.

The antioxidant activity of GL‐EO and *Od*‐EO was evaluated using DPPH and ABTS radical scavenging assays. *Od*‐EO exhibited a significantly (*p* < 0.05) higher antioxidant capacity than GL‐EO in both assays. In the DPPH assay, *Od*‐EO showed a lower IC₅₀ value (1456.95 μg/mL) compared to GL‐EO (2399.11 μg/mL). A more pronounced difference was observed in the ABTS assay, where *Od*‐EO had an IC₅₀ of 565.83 μg/mL, while GL‐EO exhibited very weak activity (IC₅₀ > 5000 μg/mL).

This enhanced activity can be mainly attributed to the high content of phenolic compounds, particularly thymol and carvacrol, present in *Od*‐EO, which are well‐known for their strong hydrogen‐donating and redox‐modulating properties (Khosravinezhad et al. 2017). Phenolic compounds exert antioxidant effects through free radical scavenging, metal ion chelation, and inhibition of oxidative chain reactions (Chen et al. 2024).

Similar findings have been reported by Esmaeili et al. (2018), who observed significantly higher antioxidant activity for *Od*‐EO compared to non‐phenolic essential oils. Although GL‐EO exhibited measurable antioxidant activity, likely due to the presence of monoterpenes such as limonene and sesquiterpenes including β‐caryophyllene (Jassal and Kaushal 2019; Aly et al. 2022), its antioxidant efficacy remained consistently lower than that of *Od‐*EO across all assay systems. This difference can be explained by the generally stronger antioxidant efficiency of phenolic compounds compared to non‐phenolic terpenoid structures.

### 4.3 Microbiological Analyses of Ground Beef

The microbiological evaluation revealed significant differences in microbial growth dynamics between treated and control ground beef samples throughout refrigerated storage. Initial total viable counts (TVC) in control samples ranged from 4.39 to 4.10 log CFU/g, and the maximum acceptable limit for fresh ground beef (7 log CFU/g) was exceeded by day 8 (6.8 log CFU/g), effectively limiting the shelf life of untreated samples to ≤ 6 days (ICMSF (International Commission on Microbiological Specifications for Foods) 2011). In contrast, GL‐EO‐treated samples maintained TVC below this critical threshold throughout the entire 10‐day storage period (Figure 3A), demonstrating superior antimicrobial efficacy compared to *Od*‐EO treatment. These findings are consistent with previous studies reporting the antimicrobial effectiveness of essential oils in meat systems (Kunová et al. 2021; Smaoui et al. 2016).

The antimicrobial activity was particularly evident against psychrotrophic aerobic bacteria, mainly Pseudomonas spp., which are recognized as the dominant spoilage microorganisms in refrigerated meat due to their proteolytic and lipolytic activities. Initial psychrotrophic total counts (PTC) on day 0 ranged from 3.52 log CFU/g in GL‐EO‐treated samples to 3.73 log CFU/g in control samples. During refrigerated storage, psychrotrophic counts in control samples increased rapidly and reached 9.87 log CFU/g by day 10. This count significantly exceeds the general acceptance limit for spoiled meat (≈7 log CFU/g; ICMSF 2011; Ercolini et al. 2010), confirming the rapid spoilage initiated by these organisms. In contrast, treated samples showed significantly lower psychrotrophic counts throughout storage (Figure 3B). The antimicrobial mechanism of these essential oils likely involves membrane disruption by bioactive compounds, as suggested by Khaledian et al. (2019) regarding the antibacterial effects of ginger essential oils phenolic diterpenes and sesquiterpenes. This is further supported by Alizadeh Behbahani and Imani Fooladi (2018), who demonstrated that psyllium mucilage combined with tarragon essential oil reduced PTC by creating a barrier to oxygen diffusion in beef products.

The GL‐EO + *Od*‐EO combination treatment demonstrated enhanced antimicrobial performance, exhibiting a greater inhibitory effect against Gram‐negative indicator microorganisms (*p* ≤ 0.05 compared with the control; Figure 3C). According to ICMSF (2011), Enterobacteriaceae counts in fresh ground meat should not exceed 2–3 log CFU/g, as higher levels indicate inadequate hygienic conditions and accelerated spoilage. In the present study, essential oil–treated samples maintained Enterobacteriaceae levels closer to this acceptable range compared to the control, confirming their antimicrobial effectiveness. Similar enhanced antimicrobial effects of combined natural preservatives have been reported in previous studies. For instance, Barkhordari and Bazargani‐Gilani (2021) demonstrated that a combined treatment of apple peel extract and zein coating enriched with ginger essential oil significantly reduced Enterobacteriaceae populations in chicken thigh meat. Similarly, Saad et al. (2019) reported enhanced efficacy for a blend of thyme, cinnamon, and garlic essential oils in minced meat. More recently, Ricardo‐Rodrigues et al. (2024) showed the concentration‐dependent activity of clove essential oil against *Escherichia coli* O157:H7 in various food matrices.

In addition to antibacterial effects, essential oil treatments significantly inhibited fungal growth during refrigerated storage (*p* ≤ 0.05 compared with the control; Figure 3D).

Fungal counts in control samples increased progressively, rising from 3.52 ± 0.24 log CFU/g on day 0 to 4.77 ± 0.08 log CFU/g by day 6, exceeding the acceptable limit for fresh meat products (3–4 log CFU/g; ICMSF, 1996). In contrast, the GL‐EO treatment significantly inhibited fungal growth, maintaining counts at 3.78 ± 0.092 log CFU/g on day 6, which remained well within the acceptable marginal range (2–4 log CFU/g). These findings demonstrate the rapid and strong antifungal efficacy of GL‐EO during refrigerated storage. Among treatments, antifungal efficacy followed the order GL‐EO > *Od*‐EO > GL‐EO + *Od*‐EO.

The reduction in fungal growth can be attributed to the antifungal bioactive compounds in the essential oils, which disrupt fungal cell membranes and inhibit spore germination. These quantitative findings align with recent studies on natural antifungal agents. For example, Gituma and Njue (2019) reported similar suppression of fungal growth in strawberries using guava leaf extract, where counts were kept below 5 log CFU/g, closely mirroring our results with GL‐EO in meat. Similar effects have been documented in other food systems (Das and Goswami 2019), supporting the potential of these essential oils for extending the shelf life of refrigerated meat products.

### 4.4 Chemical Analyses of Ground Beef

The pH dynamics of ground beef samples provided insight into preservation efficacy. The initial pH of fresh meat was 6.23, consistent with values reported in previous studies (Bazargani‐Gilani et al. 2015; Alizadeh Behbahani et al. 2017). During 12 days of storage at 4°C, control samples showed a pronounced increase in pH due to microbial metabolism and proteolytic activity leading to the accumulation of alkaline compounds such as ammonia. The results demonstrated a strong correlation between microbial growth and pH variation during storage. The initial pH decrease was associated with the early rise in total viable counts (TVC), indicating that microbial metabolism, particularly the formation of organic acids by early colonizers such as lactic acid bacteria, contributed to the initial acidification. The slight pH drop observed around day 4 in some samples aligns with this initial metabolic activity. As microbial proliferation progressed, especially in control samples, proteolytic activity intensified, leading to the accumulation of alkaline nitrogenous compounds (e.g., ammonia, trimethylamine) and a corresponding pH increase. This alkalinization further favored the growth of spoilage‐associated bacteria, establishing a positive feedback loop between proteolysis and pH elevation (Figures 3A and 5A). In contrast, treated samples exhibited significantly slower pH increases, with the GL‐EO + *Od*‐EO combination maintaining the most stable pH profile. These trends correlated well with reduced mesophilic and psychrotrophic bacterial counts, confirming the inhibitory effects of essential oils on spoilage‐related microorganisms (Khaledian et al. 2019; Elaksiry et al. 2024). This direct correlation underscores that the essential oils primary mode of action in stabilizing pH was through the effective suppression of microbial growth throughout the storage period.

Thiobarbituric acid reactive substances (TBARS), reflecting secondary lipid oxidation products, showed significant differences among treatments during refrigerated storage. Initial TBARS values in control samples ranged from 0.07 to 1.36 mg MDA/kg. By day 8 of storage, samples treated with GL‐EO (1.05 mg MDA/kg), *Od*‐EO (0.78 mg MDA/kg), and their combination (0.71 mg MDA/kg) exhibited significantly lower TBARS values, with the combined treatment showing the greatest inhibition of lipid oxidation. Control samples exceeded the widely used sensory threshold for detecting rancidity in meat during storage (≈1.0–2.0 mg MDA/kg) toward the end of storage, indicating advanced oxidative deterioration (Shah et al. 2014; Domínguez et al. 2019). The enhanced oxidative stability observed in essential oil–treated samples can be attributed to the antioxidant activity and combined effects of phenolic compounds present in the essential oils (Abdou et al. 2018). Similar inhibition of lipid oxidation in meat systems treated with plant extracts and essential oils has been reported previously (Tran et al. 2020; Shokri et al. 2020).

Total volatile basic nitrogen (TVBN), an important indicator of protein degradation and microbial spoilage, increased progressively in all samples during refrigerated storage. The control samples exceeded the accepted freshness limit of 20–25 mg/100 g (ICMSF 2011; Pearson 1968), reaching 41.44 mg/100 g by day 10, which indicates advanced spoilage. In contrast, samples treated with the combination of GL‐EO + *Od*‐EO maintained TVBN within this acceptable limit (< 20 mg/100 g) for a significantly longer period, demonstrating a clear preservative effect.

The sharp increase in TVBN observed in control samples showed a direct positive correlation with higher microbial counts (Figure 3A,C), reflecting intensified microbial deamination activity and the formation of volatile nitrogenous compounds. Conversely, the significantly lower TVBN levels in treated samples can be directly attributed to the effective suppression of proteolytic and deaminating spoilage microorganisms, as evidenced by the lower total viable and Enterobacteriaceae counts. By inhibiting these microbes, the essential oils reduced the production of volatile basic nitrogenous compounds, thereby delaying protein degradation (Hassanin et al. 2017; Zargar et al. 2016).

### 4.5 Sensory Analyses

Sensory evaluation of ground beef samples during refrigerated storage revealed that all attributes (odor, color, texture, and overall acceptability) were significantly influenced by both treatment type and storage time (*p* < 0.05). The control group exhibited rapid sensory deterioration, with odor and overall acceptability scores falling below the acceptability threshold (3) by day 6 (2.93 ± 0.11 and 3/46 ± 0.41, respectively), and texture becoming unacceptable by day 10 (2.17 ± 0.30).

In contrast, all essential oil‐treated samples maintained acceptable sensory scores throughout the 10‐day storage period. Among individual treatments, GL‐EO showed superior preservation of odor (4.23 ± 0.20 on day 6) and color (3.87 ± 0.15), which can be attributed to its high content of limonene and β‐caryophyllene, compounds known for their antioxidant activity and ability to delay myoglobin oxidation (Jassal and Kaushal 2019; Aly et al. 2022).

The combined treatment (GL‐EO + *Od‐*EO) demonstrated the highest overall acceptability, maintaining a score of 3.95 ± 0.21 on day 10, significantly higher than the control (2.33 ± 0.25) and individual treatments. This enhanced sensory preservation is likely due to the complementary antimicrobial and antioxidant effects of the two essential oils. These effects collectively delayed microbial proteolysis and lipid oxidation—key factors in texture softening and off‐odor development (Lite et al. 2020; Ghani et al. 2018). However, it is important to note that the enhanced or additive effects observed in the combined essential oils should not be interpreted as evidence of synergy, as no formal interaction assay (checkerboard or FICI) was performed.

The observed preservation of texture may be associated with previously reported mechanisms of essential oils, including their potential influence on proteolytic enzyme activity, although such effects were not directly evaluated in the present study (Ghani et al. 2018).

These findings are consistent with those of Leite et al. (2022), who reported that a combination of copaiba and oregano essential oils improved the sensory properties of fresh ground meat, with overall acceptability scores ranging from 4.1 to 4.5 during refrigerated storage. Similarly, Smaoui et al. (2016) demonstrated that essential oil‐treated minced beef maintained acceptable sensory quality for up to 9 days, supporting the shelf‐life extension observed in the present study.

The strong correlation between sensory scores and microbiological (Figure 3) and chemical (Figure 5) indicators confirms that the preservation of sensory quality in treated samples is directly linked to the inhibition of spoilage microorganisms and oxidative processes. The combined application of the oils improved texture and overall acceptability.

The concentrations applied were effective without causing sensory rejection and fall within ranges considered for natural food additives. However, further studies are required to optimize application strategies, address region‐specific regulatory requirements, and assess industrial‐scale feasibility.

## 5 Conclusion

This study demonstrated that guava leaf essential oil (GL‐EO) and *Oliveria decumbens* essential oil (*Od*‐EO) effectively preserved the quality of ground beef during refrigerated storage, extending shelf‐life by 4 days compared to control (6 days vs. 10 days). Both individual and combined treatments significantly inhibited microbial growth, delayed lipid oxidation, and maintained chemical stability, with the GL‐EO + *Od*‐EO combination showing the most pronounced preservative effects compared to individual treatments. Notably, essential oil treatments did not negatively affect sensory attributes; rather, the combined treatment enhanced texture and overall acceptability compared with the control samples. The antimicrobial and antioxidant activities of these essential oils, particularly thymol, carvacrol, limonene, and β‐caryophyllene, effectively suppressed spoilage microorganisms (*Pseudomonas* spp. and Enterobacteriaceae) and inhibited proteolytic enzyme activity, thereby preserving meat structure and freshness. These findings were consistent with previous studies on plant‐derived preservatives and highlighted the potential of GL‐EO and *Od*‐EO as natural alternatives to synthetic additives in meat products. For industrial applications, further optimization of oil concentrations and delivery strategies (e.g., encapsulation) could improve efficacy and sensory compatibility. Future studies should investigate the applicability of these.

## Author Contributions

**Porandokht Barkhordari:** software, formal analysis, data curation, investigation, writing – original draft, visualization, validation. **Ali Heshmati:** data curation, software, formal analysis, validation, visualization, methodology. **Siavash Maktabi:** conceptualization, writing – review and editing, funding acquisition, resources, supervision, project administration, methodology. **Maryam Ghaderi Ghahfarokhi:** visualization.

## Funding

This research was financially supported by the Research Council of Shahid Chamran University of Ahvaz, Iran (Grant No. SCU.VF 1403.534).

## Consent

The authors declare their consent to publish this article.

## Conflicts of Interest

The authors declare no conflicts of interest.

## Data Availability

The data that support the findings of this study are available on request from the corresponding author. The data are not publicly available due to privacy or ethical restrictions.
